# Remote Sensing Extraction Method of Tailings Ponds in Ultra-Low-Grade Iron Mining Area Based on Spectral Characteristics and Texture Entropy

**DOI:** 10.3390/e20050345

**Published:** 2018-05-06

**Authors:** Baodong Ma, Yuteng Chen, Song Zhang, Xuexin Li

**Affiliations:** 1 Key Laboratory of Ministry of Education on Safe Mining of Deep Metal Mines, Northeastern University, Shenyang 110819, China; 2 Institute for Geoinformatics & Digital Mine Research, Northeastern University, Shenyang 110819, China

**Keywords:** spectral characteristics, texture, entropy, ultra-low-grade iron, tailings pond, Landsat 8 OLI image

## Abstract

With the rapid development of the steel and iron industry, ultra-low-grade iron ore has been developed extensively since the beginning of this century in China. Due to the high concentration ratio of the iron ore, a large amount of tailings was produced and many tailings ponds were established in the mining area. This poses a great threat to regional safety and the environment because of dam breaks and metal pollution. The spatial distribution is the basic information for monitoring the status of tailings ponds. Taking Changhe Mining Area as an example, tailings ponds were extracted by using Landsat 8 OLI images based on both spectral and texture characteristics. Firstly, ultra-low-grade iron-related objects (i.e., tailings and iron ore) were extracted by the Ultra-low-grade Iron-related Objects Index (ULIOI) with a threshold. Secondly, the tailings pond was distinguished from the stope due to their entropy difference in the panchromatic image at a 7 × 7 window size. This remote sensing method could be beneficial to safety and environmental management in the mining area.

## 1. Introduction

In the early Twenty-First Century, with the rapid development of the iron and steel industry, the supply of iron ore was intense in the international market [1,2]. In China, the iron resource is abundant, but high-grade ores are scarce. In this context, a new type of ultra-low-grade iron appeared with the development and utilization of China’s iron ore resources [3,4]. The ultra-low-grade magnetite, with a lower than 20% total iron content, has been extracted by open-pit mining and processed massively since 2001 in Hebei Province, China [5,6]. Changhe Mining Area is an important mining area in the region and has proven reserves of more than 2.3 billion tons of ultra-low-grade iron ore [7].

Because the ratio of the concentration (the number of tons of ore required to produce one ton of concentrate) is too high (about 10), a large amount of tailings is produced [8]. Tailings ponds may result in a serious safety threat (e.g., dam breaking) [9] and environmental pollution (e.g., dust pollution) [10]. How to obtain the spatial distribution of tailings pond quickly and accurately is of great significance to guide the regional environmental governance, ecological restoration and production safety monitoring [11,12,13]. However, because of the relatively short development time of this type of iron ore, the remote sensing methods to extract the above information are lacking.

To extract thematic information, many remote sensing indices were designed for describing targets based on their spectral characteristics. For example, the Normalized Difference Vegetation Index (NDVI), the Normalized Difference Built-up Index (NDBI) and the Normalized Difference Water Index (NDWI) were designed respectively for extraction of vegetation, built-up areas and water bodies [14,15,16]. However, sometimes, the spectral characteristics alone are not sufficient for the exact extraction of targets. Texture characteristics could play an important role in remote sensing interpretation [17,18]. Based on both spectral and texture characteristics, remote sensing images could be used to extract the information of the object of interest. The Gray Level Co-occurrence Matrix (GLCM) is a widely-used method for the statistical analysis of texture [19,20]. Entropy is one of the commonly-used grayscale symbiotic matrices of GLCM and has been proven to be effective for the interpretation remote sensing data [21,22,23]. Thus, this paper uses entropy to measure the texture features of the tailings.

Therefore, the purpose of this paper is to research the spectral and texture characteristics of tailings based on the field spectra and satellite remote sensing data and then integrate two characteristics to extract tailings ponds.

## 2. Materials

### 2.1. Study Area

**Figure 1:**
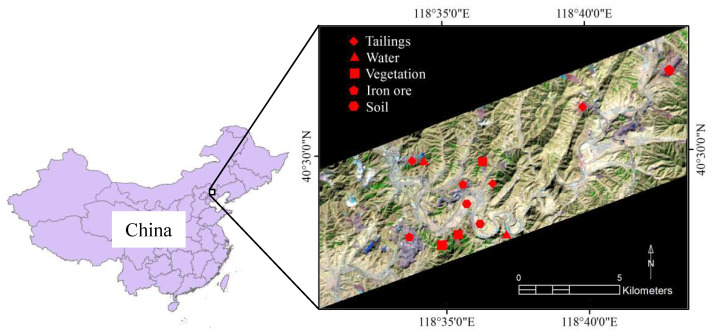
Location of the Changhe Mining Area (the right is the false-color-composite image, Landsat 8 OLI 654).

The study area, called Changhe Mining Area (about 134 km^2^), is located in Kuancheng County, Hebei Province, China (Figure 1). The study area is estimated to have 2.7 billion tons of iron ore, which accounts for one third of the magnetite reserves in Hebei Province [7]. At present, annual production of iron powder is more than 10 million tons in this area. This area is covered by dense vegetation and has an average elevation of 300–500 m with a mountainous landform. Changhe River is the main river in this area. It has a continental monsoon climate with an annual precipitation of 662.5 mm and an annual temperature of 8.7 °C.

### 2.2. Field Spectral Data

Vegetation canopy, bare soil, water body, iron ore and iron tailings were measured respectively with the HR-1024 spectrometer (American Spectra Vista Corporation (SVC)). The SVC spectrometer covered a spectral range of 350–2500 nm with a varying spectral resolution of 3.5 nm for 350–1000 nm, 9.5 nm for 1000–1890 nm and 6.5 nm for 1890–2500 nm. The measurements were carried out between 10:00 a.m. and 2:00 p.m. on sunny days from 10–15 March 2016. For each feature class, spectra were measured on two or three plots in the study area (shown in Figure 1). There were 5–10 spectral samples of each plot. The spectral curves of these typical objects were processed by SVC software. Then, the average spectra of these samples were set as the spectra of each feature class.

### 2.3. Remote Sensing Data

The Landsat 8 OLI image (Path 122 and Row 32) was selected for the iron tailings detection (downloaded from USGS, http://glovis.usgs.gov/). Its spatial resolution was 30 m in multi-spectral bands and 15 m in the panchromatic band [24]. The image was acquired on 10 March 2016. The image was geometrically registered to the high-resolution GeoEye image. Then, the image was atmospherically corrected to surface reflectance by using the Fast Line-of-Slight Atmospheric Analysis of Spectral Hypercubes (FLAASH) module of the ENVI software [25]. The error of atmospheric correction was 0.03 by referencing the field spectral measurement of ground samples, and the error of the geometrical registration was lower than one pixel (i.e., 30 m).

High-resolution GeoEye image, with a resolution of approximate 0.4 m, was selected as the ancillary data (download from GoogleEarth). It was acquired on 13 September 2015. Using the image, we could draw boundaries of tailings ponds for the validation of the Landsat result.

## 3. Methods

**Figure 2:**
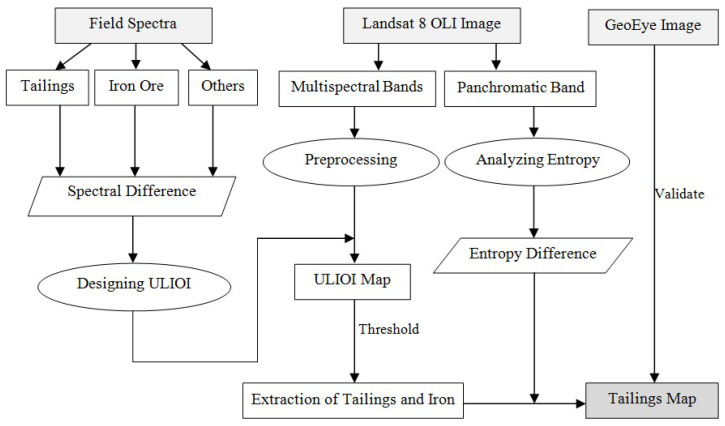
A flowchart for detecting tailings pond in the mining area. ULIOI, Ultra-Low-Grade Iron-Related Objects Index.

In general, field spectra were used to design the index for extraction of tailings and iron ore. Then, the Landsat 8 OLI image, including multispectral bands and the panchromatic band, was used to detect tailings ponds. The result was validated based on the GeoEye image (Figure 2).

### 3.1. Extraction Method of Ultra-Low-Grade Iron-Related Objects Based on Spectral Characteristics

#### 3.1.1. Spectral Characteristics of Tailings Pond

**Figure 3:**
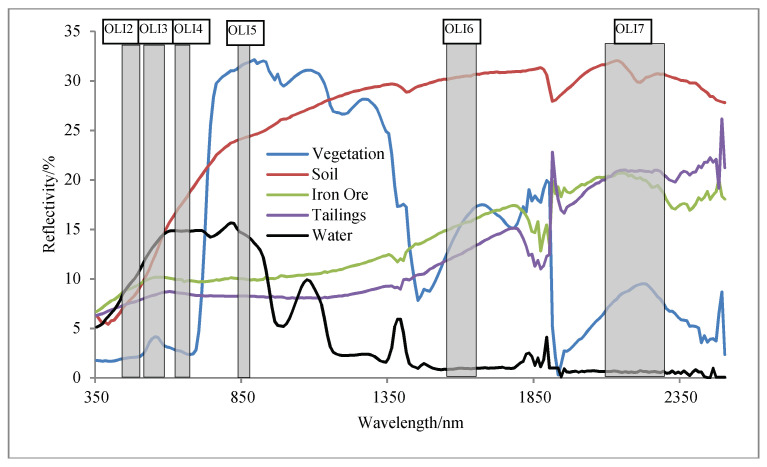
A comparison of spectral curve of different objects (i.e., vegetation, soil, iron ore, tailings, and water).

According to a chemical test, it was found that the tailings contained approximate 10% TFe, 45% SiO_2_, 15% CaO, 10% MgO and 10% Al_2_O_3_. The oxidized Fe^3+^ would lead to an absorption peak near 900 nm [26]. Therefore, compared with other objects, the reflectivity of the tailings in the green band (OLI 3) was nearly equal to that in the red band (OLI 4), and the reflectivity of the tailings in the SWIR2 band (OLI 7) was greater than that in the SWIR1 band (OLI 6) (Figure 3). However, the spectral curves of tailings and iron ore were similar to each other. Thus, the tailings pond and the stope could not be distinguished in the multi-spectral bands.

#### 3.1.2. Design of Ultra-Low-Grade Iron-Related Objects Index

In this paper, tailings and iron ore in the mining area are called ultra-low-grade iron-related objects. Because tailings and iron ore have similar spectral characteristics, an Ultra-low-grade Iron-related Objects Index (ULIOI) was designed to extract these two objects based on their spectral characteristics. OLI Bands 3, 4, 6 and 7 were selected to construct the ULIOI. The formula is as follows.

(1) $$\mathrm{ULIOI}=\frac{\mathrm{OLI}3\cdot \mathrm{OLI}7}{\mathrm{OLI}4\cdot \mathrm{OLI}6}$$

where OLI3, OLI4, OLI6 and OLI7 represent the reflectivity of objects in OLI Bands 3, 4, 6 and 7, respectively.

### 3.2. Distinguishing Tailings Pond from Stope Based on Texture Characteristics

GLCM is a method to describe texture by studying the spatial correlation property of gray. It is a statistical description of a local area, or the whole area of the image, or a matrix of two pixels within a certain distance. The element values in the matrix represent the joint conditional probability density between the gray levels. Its formula is as follows:

(2) p(i,j,d,θ)={[(x,y),(x+a,y+b)|f(x,y)=i;f(x+a,y+b)=j]}

where p is the joint conditional probability density, d is the given distance from the starting point (x, y) to the end point (x + a, y + b), i is the gray level of the starting point, j is the gray level of the end point and θ is the direction from the starting point to the end point. In this paper, the four most common directions (0°, 45°, 90° and 135°) are used in the calculation of GLCM, respectively.

Entropy is the representation of the complexity of the spatial relationship of the image. Its formula is as follows:

(3) $$E=-\sum_{i}\sum_{j}p\left(i,\;j\right)\log\left(p\left(i,\;j\right)\right)$$

where E is the entropy and p(i, j) represents the probability values in the GLCM. When the type of image is complex or the texture of the image is rough, the value of the entropy will be very great. That is, the more complex the object is, the greater the entropy is. In addition, to compare the difference of the entropy calculated for different processing window sizes, the window size is set as 3 × 3, 5 × 5, 7 × 7, 9 × 9 and 11 × 11.

## 4. Results and Discussion

### 4.1. Extraction of Ultra-Low-Grade Iron-Related Objects

**Figure 4:**
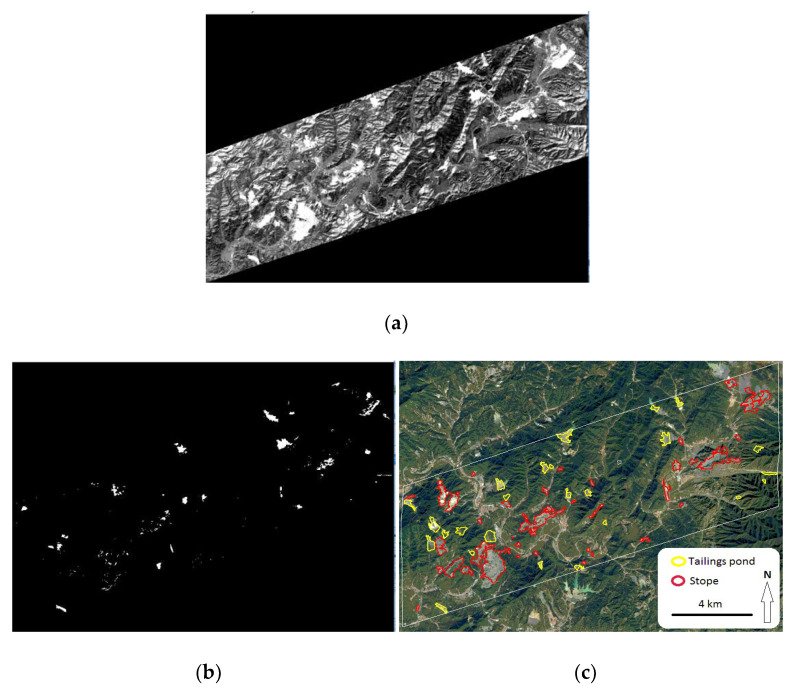
Extraction of ultra-low-grade iron-related objects based on Ultra-low-grade Iron-related Objects Index (ULIOI). (**a**) ULIOI image; (**b**) ultra-low-grade iron-related objects (in white color) extracted with the threshold of 1.07; (**c**) high-resolution GeoEye image.

In the ULIOI map, the ultra-low-grade iron-related objects were prominent, and other objects (vegetation, bare soil and water body) were suppressed (Figure 4a). Thus, the grayscale differences between the two kinds of objects were significantly widened. With the help of high-resolution GeoEye image, 100 pixels of iron-related objects were selected to determine the threshold in the ULIOI image. From these pixels, the mean value (μ) of ULIOI was 1.12 and the standard deviation value (σ) of OLIOI was 0.025. Then, μ ± 2σ could be set as the threshold. Because iron-related objects were highlighted in the ULIOI image, only the lower limit was needed for the extraction. Thus, μ − 2σ, the value 1.07 in the study, was set as the threshold to extract iron-related objects (Figure 4b). After verification by the use of the high-resolution GeoEye image, it was found that the overall accuracy based on the error matrix was 93.14% (Figure 4c). However, the tailings pond was mixed with the stope.

ULIOI is a ratio index, which is sensitive to atmospheric effects. Therefore, the image should be atmospherically corrected before it is used for calculating ULIOI. Due to the calculation being based on an atmospherically-corrected image, it could be used universally for different study areas and satellite scenes. In addition, the high-resolution GeoEye image acquired on 13 September 2015 was selected to validate the Landsat (acquired on 10 March 2016) result. Fortunately, the boundary of iron-related objects was nearly changeless between the two dates because most companies in this area had been asked to restrict the mining of iron ore for air pollution control since the beginning of 2015. However, the two images should be acquired at the same time if possible. Therefore, the boundary would hardly change in the acquiring interval between GeoEye and Landsat images.

### 4.2. Extraction of Tailings Pond

**Figure 5:**
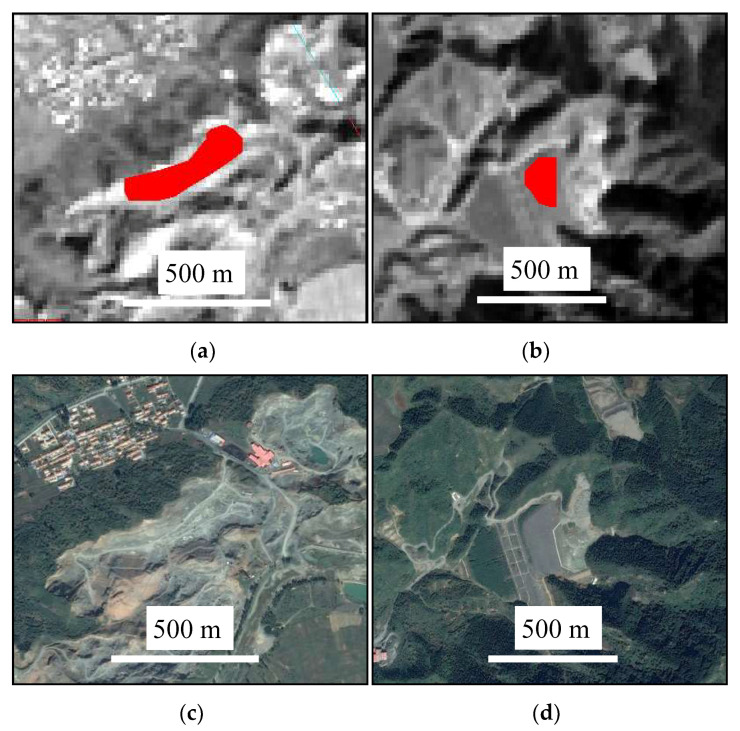
The tailings pond and stope for comparison of the entropy value. (**a**) Stope in the panchromatic image (entropy was calculated in the red-color area); (**b**) tailings pond in the panchromatic image (entropy was calculated in red-color area); (**c**) stope in the GeoEye image; (**d**) tailings pond in the GeoEye image.

Entropy was calculated in a typical tailings pond and a typical stope, respectively, using on the panchromatic band (Figure 5). These entropy values in different directions and different processing window sizes are shown in Table 1. Generally, for both the tailings pond and stope, the entropy increased with the increasing size of the processing window. In addition, the entropy of the tailings pond was smaller than that of the stope, which indicates that the image of the stope was more complex than that of the tailings pond, or the texture of the stope image was rougher.

Concretely, for the tailings pond, the entropy in the 90° direction was minimum. This was related to the direction of the tailings pond. The tailings pond ran approximately east-west, and the tailings stacked in the pond ran approximately north-south (the 90° direction). Thus, the texture of the tailings image in the 90° direction was finer than other directions. However, for the stope, there was no significant difference in entropy among different directions. In other words, the texture of the iron ore in the stope had no obvious directional characteristics.

**Figure 6:**
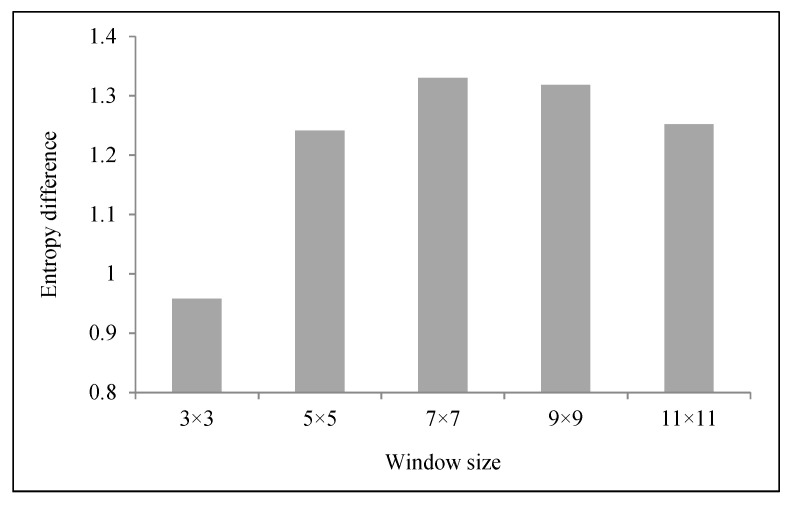
The entropy difference between the tailings pond and stope in different processing window sizes.

A threshold value of entropy was used for distinguishing tailings pond from stope. Firstly, a 7 × 7 processing window of entropy was selected as the window size because the entropy difference between tailings pond and stope was greatest at a 7 × 7 window size (Figure 6). Secondly, the texture direction was determined. For the stope, 0° was selected as the texture direction because there was no significant difference in the entropy among different directions. For the tailings pond, because the running direction of these tailings ponds was various in the mining area, 0° was also selected as the texture direction. In short, 0° was selected as the texture direction for both tailings ponds and stopes. At last, the value of 2.4 (the approximate mean value of the two entropy values) was set as the threshold value of entropy in the 0° direction and a 7 × 7 window to distinguish tailings ponds from stopes.

**Figure 7:**
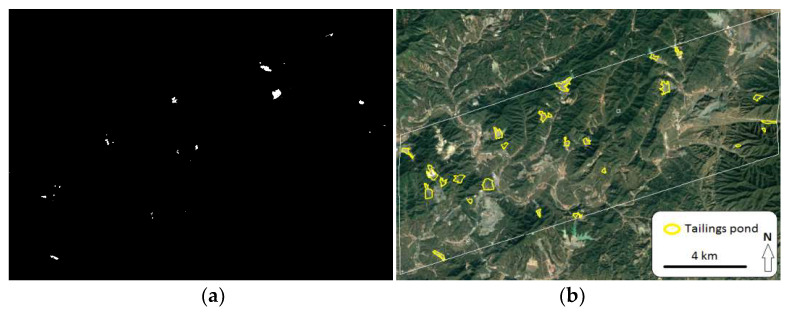
Extraction of tailings ponds based on entropy difference. (**a**) Tailings ponds (in white color) extracted with the threshold of 2.4; (**b**) high-resolution GeoEye image.

Based on the extracted ultra-low-grade iron-related objects, the pixels with an entropy value lower than 2.4 were classified as tailings ponds (Figure 7). Most tailings ponds were extracted, and the overall accuracy of the extraction was 91.30%. However, some tailings ponds after reclamation were not extracted because their surfaces were covered with vegetation. Those tailings ponds after reclamation would hardly produce environmental pollution and safety problems. Thus, the extraction result would make sense for regional environmental governance and ecological restoration.

## 5. Conclusions

Due to the huge amount of ultra-low-grade iron beneficiation, fast detection and accurate extraction of tailings are of great significance to the regional environment’s protection and safety management. Using field spectral data and satellite remote sensing image data, this study realized the extraction of tailings ponds. The main conclusions were drawn through this study as follows.

(1) Based on spectral characteristics, ULIOI was designed to extract ultra-low-grade iron-related objects (i.e., tailings and iron ore) by using Landsat 8 OLI multi-spectral Bands 3, 4, 6 and 7. The extraction was realized with a threshold. This was the basis for the extraction of tailings ponds.
(2) Based on texture characteristics, entropy was calculated to distinguish tailings ponds from stopes by using a Landsat 8 OLI panchromatic image. Texture characteristics of tailings ponds were more consistent, and the entropy value was smaller. Texture characteristics of the stope were more complicated, and the entropy value was greater. The entropy value in a 7 × 7 window size was selected to differentiate tailings ponds and stopes with the threshold of 2.4.

The extraction could provide spatial distributing information of tailings ponds in the ultra-low-grade iron mining area. Due to the extensive development of iron ore both in area and production, it would have a good effect on the environmental and safety management in mining areas.

## Figures and Tables

**Table 1:** The entropy value of the tailings pond and stope in different directions (i.e., 0°, 45°, 90° and 135°) and different processing window sizes (i.e., 3 × 3, 5 × 5, 7 × 7, 9 × 9 and 11 × 11).

Window Size	Tailings Pond				Stope			
	0°	45°	90°	135°	0°	45°	90°	135°
3 × 3	0.983	1.240	0.941	0.980	1.941	1.981	1.969	1.961
5 × 5	1.500	1.651	1.389	1.520	2.741	2.785	2.780	2.777
7 × 7	1.883	1.897	1.740	1.905	3.213	3.257	3.262	3.307
9 × 9	2.213	2.163	2.066	2.255	3.531	3.595	3.606	3.686
11 × 11	2.514	2.434	2.380	2.577	3.766	3.844	3.871	3.978

## References

[1] Sonter L.J., Barrett D.J., Soares-Filho B.S., Moran C.J. (2014). Global demand for steel drives extensive land-use change in Brazil’s Iron Quadrangle. Glob. Environ. Chang..

[2] Yellishetty M., Ranjith P.G., Tharumarajah A. (2010). Iron ore and steel production trends and material flows in the world: Is this really sustainable?. Resour. Conserv. Recycl..

[3] Li H.-M., Li L.-X., Yang X.-Q., Cheng Y.-B. (2015). Types and geological characteristics of iron deposits in China. J. Asian Earth Sci..

[4] Zhang Z., Hou T., Santosh M., Li H., Li J., Zhang Z., Song X., Wang M. (2014). Spatio-temporal distribution and tectonic settings of the major iron deposits in China: An overview. Ore Geol. Rev..

[5] Shuaixing S., Ming Z., Ming T., Maohe L. (2015). Recovery of phosphorite from coarse particle magnetic ore by flotation. Int. J. Miner. Process..

[6] Li L.X., Li H.M., Wang D.Z., Liu M.J., Yang X.Q., Chen J. (2012). Ore genesis and ore-forming age of the Tiemahabaqin ultra-low-grade iron deposit in Chengde, Hebei Province, China. Rock Miner. Anal..

[7] Ma B., Pu R., Wu L., Zhang S. (2017). Vegetation Index Differencing for Estimating Foliar Dust in an Ultra-Low-Grade Magnetite Mining Area Using Landsat Imagery. IEEE Access.

[8] Cui L., Zhou D. (2015). Environmental problems caused by ultra-low-grade magnetite exploitation and countermeasures. Resour. Environ. Eng..

[9] Coulibaly Y., Belem T., Cheng L. (2017). Numerical analysis and geophysical monitoring for stability assessment of the Northwest tailings dam at Westwood Mine. Int. J. Min. Sci. Technol..

[10] Stovern M., Rine K., Russell M., Felix O., King M., Saez A., Betterton E. (2015). Development of a dust deposition forecasting model for mine tailings impoundments using in situ observations and particle transport simulations. Aeolian Res..

[11] Hu X., Oommen T., Lu Z., Wang T., Kim J.-W. (2017). Consolidation settlement of Salt Lake County tailings impoundment revealed by time-series InSAR observations from multiple radar satellites. Remote Sens. Environ..

[12] Mohamed M.H., Wilson L.D., Headley J.V., Peru K.M. (2008). Novel materials for environmental remediation of tailing pond waters containing naphthenic acids. Process Saf. Environ. Prot..

[13] Zornoza R., Faz Á., Carmona D.M., Acosta J.A., Martínez-Martínez S., de Vreng A. (2013). Carbon mineralization, microbial activity and metal dynamics in tailing ponds amended with pig slurry and marble waste. Chemosphere.

[14] Jucker Riva M., Daliakopoulos I.N., Eckert S., Hodel E., Liniger H. (2017). Assessment of land degradation in Mediterranean forests and grazing lands using a landscape unit approach and the normalized difference vegetation index. Appl. Geogr..

[15] Liou Y.-A., Nguyen A.K., Li M.-H. (2017). Assessing spatiotemporal eco-environmental vulnerability by Landsat data. Ecol. Indic..

[16] Jawak S.D., Luis A.J. (2015). A Rapid Extraction of Water Body Features from Antarctic Coastal Oasis Using Very High-Resolution Satellite Remote Sensing Data. Aquat. Procedia.

[17] Schmid T., Koch M., DiBlasi M., Hagos M. (2008). Spatial and spectral analysis of soil surface properties for an archaeological area in Aksum, Ethiopia, applying high and medium resolution data. CATENA.

[18] Muller S.J., van Niekerk A. (2016). Identification of WorldView-2 spectral and spatial factors in detecting salt accumulation in cultivated fields. Geoderma.

[19] Su H., Wang Y., Xiao J., Li L. (2013). Improving MODIS sea ice detectability using gray level co-occurrence matrix texture analysis method: A case study in the Bohai Sea. ISPRS J. Photogramm. Remote Sens..

[20] Roberti de Siqueira F., Robson Schwartz W., Pedrini H. (2013). Multi-scale gray level co-occurrence matrices for texture description. Neurocomputing.

[21] Pourghasemi H.R., Mohammady M., Pradhan B. (2012). Landslide susceptibility mapping using index of entropy and conditional probability models in GIS: Safarood Basin, Iran. CATENA.

[22] Han B., Wu Y. (2017). A novel active contour model based on modified symmetric cross entropy for remote sensing river image segmentation. Pattern Recognit..

[23] Padmanaban R., Bhowmik A.K., Cabral P., Zamyatin A., Almegdadi O., Wang S. (2017). Modelling Urban Sprawl Using Remotely Sensed Data: A Case Study of Chennai City, Tamilnadu. Entropy.

[24] Roy D.P., Kovalskyy V., Zhang H.K., Vermote E.F., Yan L., Kumar S.S., Egorov A. (2016). Characterization of Landsat-7 to Landsat-8 reflective wavelength and normalized difference vegetation index continuity. Remote Sens. Environ..

[25] (2009). FLAASH User’s Guide, Atmospheric Correction Module: QUAC and FLAASH User’s Guide.

[26] Xu A., Ma B., Li X., Wu L. (2017). Spectral testing and quantitative inversion for dust of iron tailings on leaf. Remote Sens. Land Resour..

